# Theranostic Gold Nanoparticles Encapsulated in a PEGylated Liposome as an Effective Radiosensitizer for Cancer Radiation Therapy

**DOI:** 10.1021/acsabm.5c00908

**Published:** 2025-08-26

**Authors:** Jinyeong Choi, Gaeun Kim, Beomjin Park, Jiwoo Park, Shengjun Li, Wooseung Lee, Miyeon Jeon, Chiwoo Oh, Sangmin Lee, Sung-Joon Ye, Hyung-Jun Im

**Affiliations:** † Department of Applied Bioengineering, Graduate School of Convergence Science and Technology, 26725 Seoul National University, Seoul 08826, Republic of Korea; ‡ Department of Molecular Medicine and Biopharmaceutical Sciences, Graduate School of Convergence Science and Technology, Seoul National University, Seoul 08826, Republic of Korea; § Cancer Research Institute, Seoul National University, 03080 Seoul, Republic of Korea; ∥ Research Institute for Convergence Science, Seoul National University, Seoul 08826, Republic of Korea; □ Advanced Institute of Convergence Technology, 26725 Seoul National University, Suwon 16229, Republic of Korea

**Keywords:** theranostics, gold nanoparticles, radiotherapy, radiosensitizer, PEGylated liposome

## Abstract

High-Z materials enhance radiation dose deposition primarily through strong photoelectric absorption. Leveraging this property, nanoparticles based on high-Z content materials can be utilized as nanoscale radiosensitizers to enhance the efficacy of radiotherapy. Notably, gold nanoparticles (Au: gold, AuNPs) have been intensively investigated due to their excellent radiosensitizing effect and straightforward synthesis process. However, without additional modifications, they suffer from unfavorable biodistribution and inefficient tumor targeting, which limit their efficacy as radiosensitizers. Herein, we developed ultrasmall AuNPs encapsulated in PEGylated liposome (PEG: polyethylene glycol, Au-Lipo) and optimized their PEGylated lipid composition to improve their performance as radiosensitizers. Au-Lipo formulation was synthesized and optimized to exhibit good stability and cellular uptake *in vitro*. Au-Lipo demonstrated a dose enhancement factor at 2 Gy (DEF_2Gy_) of 2.56 which tends to have higher value compared to the commercial AuNPs, AuroVist. Furthermore, Au-Lipo showed substantial tumor uptake in positron emission tomography with significantly improved tumor therapeutic efficacy compared to radiotherapy alone in 4T1 tumor-bearing mice (*P* = 0.035). These results suggest that Au-Lipo is a promising radiosensitizing agent, offering systemic injectability, enhanced tumor-targeting efficiency, and significant therapeutic potential for improving radiotherapy outcomes.

## Graphical abstract

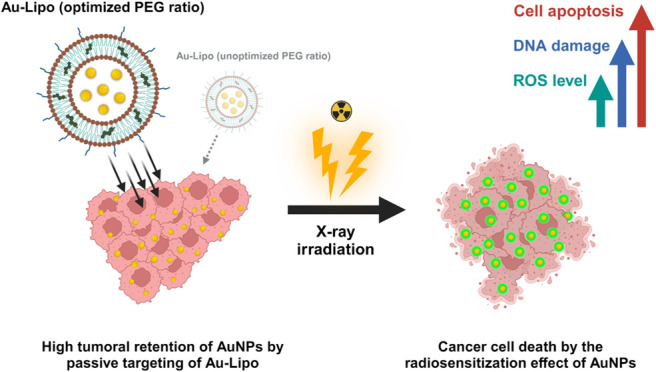

## Introduction

Radiotherapy is one of the primary modalities for cancer treatment, with approximately 50–60% of cancer patients receiving this intervention at some point during their treatment, either for therapeutic or palliative purposes.^1–3^ Radiotherapy uses ionizing radiation including X-rays, γ rays, and high energy particles.^4^ Cancer tissue exhibits greater vulnerability to radiation compared to normal cells due to its propensity for continuous division rather than effective DNA repair.^5–7^ Despite the heightened sensitivity of cancer cells to radiation, surrounding normal cells may still incur damage, presenting a significant challenge of nonspecific harm.^8,9^

Radiosensitizer, a substance that makes cancer cells more sensitive to radiation therapy, has been extensively studied to overcome the limitations of radiotherapy.^10^ High-Z materials based nanoparticles exhibit a physical dose enhancement effect as well as subsequent biological effects, making them promising radiosensitizers. Specifically, the dominant photoelectric effect in high-Z materials generates photoelectrons, secondary photons, and Auger electrons leading to localized radiation dose enhancement.^11–13^ Additional biological mechanisms of dose enhancement contain oxidative stress,^14^ DNA damage,^15^ cell cycle effect,^16^ and bystander effect.^17,18^

AuNPs are the most studied high-Z material-based nanoparticle having various useful characteristics for cancer theranostics with biocompatibility. AuNPs can be made into a desired size and shape easily, and their characteristics might be different by the size and shape.^19,20^

Advantageous characteristics of AuNPs, revealed in the context of biomedical applications, include surface plasmon resonance for biosensors,^21^ optical properties,^22^ photothermal property,^23^ potential for imaging agent, and radiosensitization.^24^ Since the first study showed that AuNPs had an X-ray dose enhancement effect in 2004,^25^ various studies on the radiosensitization effect of gold nanoparticles have been reported actively.^26–29^ Many reports concluded that smaller AuNPs will be more effective to kill cancer cells because it can be more possible to localize nearby nucleus than larger AuNPs.^30,31^ Also, ultrasmall AuNPs are quickly cleared from the body by kidney clearance.^32^ Fast kidney clearance of ultrasmall AuNPs gives a lower toxicity; however, it can limit tumor-targeting efficiency simultaneously. In this context, it is required to maximize the therapeutic efficacy of AuNPs by optimizing the delivery platform for tumor targeting.

In this study, we developed PEGylated liposomes encapsulating ultrasmall AuNPs (PEG: poly­(ethylene glycol), Au-Lipo) to enhance blood circulation and improve passive tumor-targeting efficiency. Additionally, optimal stability and cellular uptake were achieved through optimization of PEGylation.

## Materials and Methods

### Materials

Gold­(III) chloride trihydrate (HAuCl_4_), sodium hydroxide (NaOH), Tetrakis hydroxymethyl phosphonium chloride (THPC), cholesterol, 1, 1′-Dioctadecyl-3, 3, 3′, 3′-Tetramethylindodicarbocyanine (DiD), 1, 1′-Dioctadecyl-3, 3, 3′, 3′-Tetramethylindotricarbocyanine iodide (DiR), Hoechst 33342, 3-[4,5- dimethylthiazol-2-yl]-2,5-diphenyltetrazolium bromide reagent (MTT) were purchased from Sigma-Aldrich (Missouri). 1,2-distearoyl-*sn*-glycero-3-phosphocholine (DSPC), 1,2-distearoyl-*sn*-glycero-3-phosphoethanolamine-N-[amino­(polyethylene glycol)-2000] (DSPE-PEG2000-amine) were obtained from Avanti Polar Lipids, Inc. (Alabama). 1,2-distearoyl-*sn*-glycero-3-phosphoethanolamine-N-[methoxy­(polyethylene glycol)-5000] (DSPE-PEG5000) was purchased from Creative PEGWorks (North Carolina). 2-(p-Isothiocyanatobenzyl)-1,4,7-triazacyclononane-N,N′,N,″-triacetic acid trihydrochloride (p-SCN-Bn-NOTA) was obtained from Futurechem (Futurechem co.,Seoul, Korea). The 4T1 cell line was purchased from the American Type Cell Collection (Virginia). Balb/c-nude mice were obtained from Orient Bio (Orient Bio Inc., Gapyeong, Korea). Cell culture medium, RPMI 1640, was purchased from Welgene (Gyeongsan, Korea). Fetal bovine serum (FBS) was obtained from Cytiva (Massachusetts). Penicillin-streptomycin (P/S), phosphate-buffered saline (PBS), was purchased from Gibco (New York). Flow cytometry kit for apoptosis analysis and γ-H2AX antibody were obtained from Abcam (Cambridge, U.K). CellROX green reagent was purchased from Invitrogen (Massachusetts). Other cell experiment accessories are obtained from SPL (Pocheon, Korea). Distilled water (DW) was prepared using a Milli-Q filtration system from MerckMillipore (Massachusetts). Chloroform, methanol, and dimethyl sulfoxide (DMSO) were purchased from Daejung Chemical & Metals Co. (Siheung, Korea). Commercial AuNP, AuroVist-1.9 nm (AuroVist), was obtained from Nanoprobes Inc. (New York).

### Instruments

An ultrasonic probe sonicator (Sonifier SFX250, BRANSON, Missouri) was used to sonicate the hydrated lipid bilayer solution. For purification, a 0.2 μm syringe filter (25CP020AS, Advantec Toyo Roshi Kaisha Ltd., Tokyo, Japan) and PD-10 column (Cytiva) was used. Synthesized liposome was characterized with a transmission electron microscope (TEM, JEM1010, JEOL, Tokyo, Japan), dynamic light scattering (DLS, ZETASIZER Nano ZS, Malvern Instrument Ltd., Worcestershire, U.K.), and nanoparticle tracking analyzer (NTA, Nanosight Pro, Malvern Instrument Ltd., Worcestershire, U.K.). Microplate reader (SYNERGY H1, BioTek, Vermont) was used to quantify the AuNPs amount. A confocal laser scanning microscope (CLSM, Nikon A1R, Nikon Co., Tokyo, Japan) and IVIS (IVIS spectrum, PerkinElmer, Massachusetts) were utilized to acquire fluorescence images from *in vitro* and *in vivo* respectively. X-ray was irradiated by using X-RAD320 (Precision Xray, Connecticut). For apoptosis analysis, flow cytometry was performed with a Guava easyCyte5 (Cytek, California). Genisys4 (Sofie Biosciences, Virginia) was utilized for positron emission tomography (PET) to analyze biodistribution of the radioisotope labeled liposome.

### Preparation of Ultrasmall AuNPs

Ultrasmall AuNPs were synthesized by sequentially adding 1.5 mL of DW, 40 μL of 0.4 M NaOH, 35 μL of 1.2% THPC, and 140 μL of 1% HAuCl_4_ solution under stirring at 500 rpm. The reaction is complete when the solution turns reddish-brown, indicating AuNPs formation.

### Au-Lipo Synthesis

DSPC, DSPE-PEG5000, and cholesterol were dissolved in a 2:1 chloroform-to-methanol solvent at a molar ratio of 6.3:1.6:2.6. After the organic solvent was evaporated to form a lipid thin film, it was rehydrated in 2 mL of ultrasmall AuNPs solution. The hydrated lipid bilayers were sonicated using an ultrasonic probe sonicator for 12 min to make an ultrasmall AuNP-loaded liposome using a self-assembly method. Liposome nanoparticles were purified using a 0.2 μm syringe filter, followed by size exclusion chromatography with a PD-10 column. For *in vitro* an *in vivo* uptake test, the Au-Lipo was synthesized with fluorescent dyes by adding DiD and DiR respectively to the lipid thin film. For the synthesis of liposome particles used in *in vivo* PET imaging, NOTA-SCN was reacted with DSPE-PEG2000-amine for 12 h. Following this, Au-Lipo was synthesized using an additional DSPE-PEG2000-NOTA lipid mixture by the same method described above. Then, ^64^Cu was reacted with Au-Lipo for 1 h at 37 °C and subsequently purified using a PD-10 column (Scheme 1.)

**Scheme 1:**
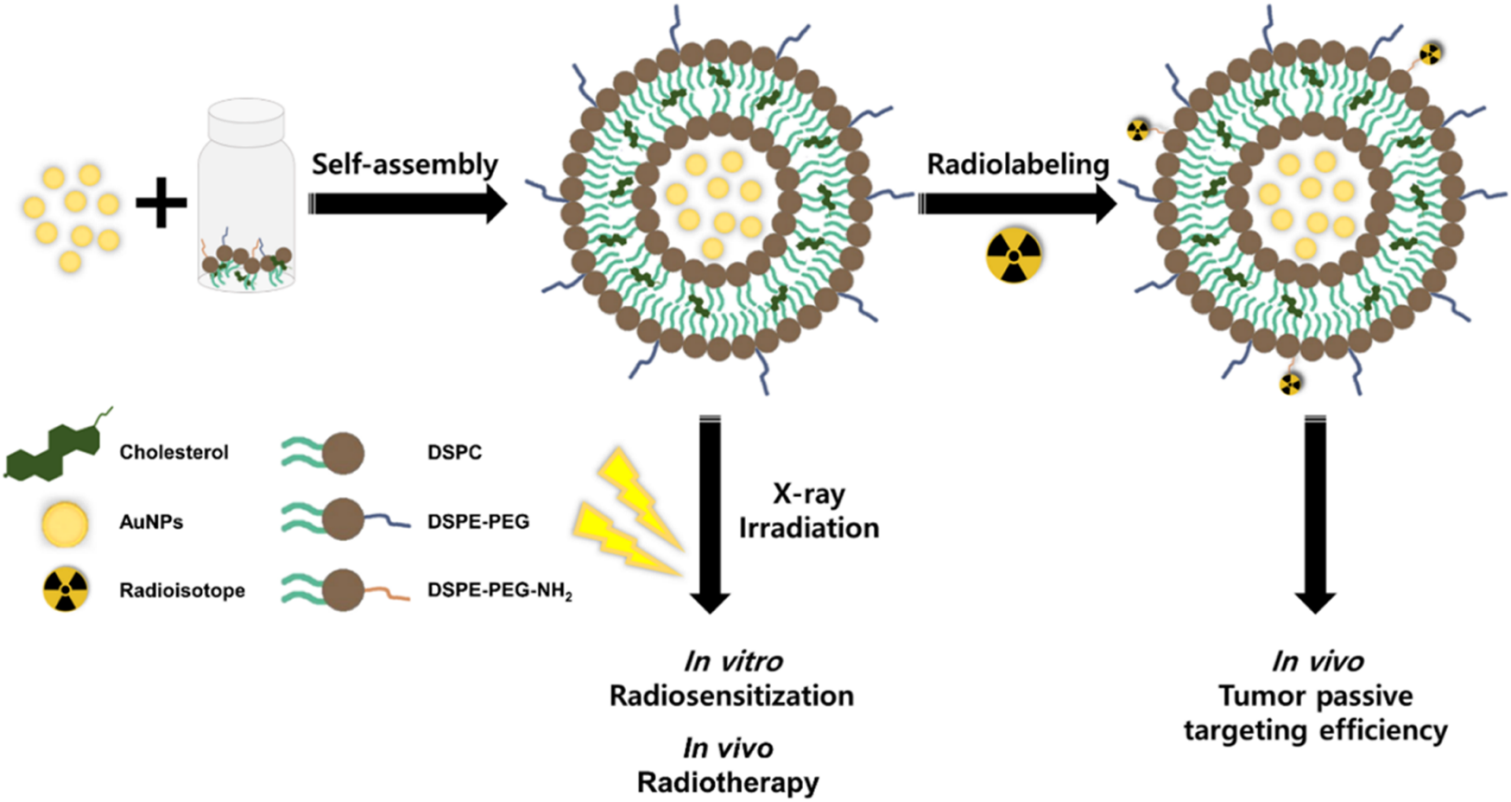
Schematic Illustration of Au-Lipo Synthesis and Experimental Procedures

### Characterization of Au-Lipo

The presence of AuNPs loaded in liposome were confirmed by TEM. Hydrodynamic size and surface charge of AuNPs and Au-Lipo were measured using DLS. AuNPs loaded in Au-Lipo was quantified using a calibration curve of AuNPs based on absorbance at 509 nm and the loading efficiency was calculated according to eq 1.

1 $$\mathrm{loading}\;\mathrm{efficiency}=\frac{W_{\mathrm{Au}‐\mathrm{Lipo}}}{W_{\mathrm{AuNPs}}}\times 100\left(\%\right)$$

where *W*_Au‑Lipo_ is the amount of AuNPs in the Au-Lipo sample and *W*_AuNPs_ is the amount of AuNPs initially added to synthesize Au-Lipo.

To assess the *in vitro* stability over time, the hydrodynamic sizes of Au-Lipo and AuroVist suspended in DW, PBS, and RPMI 1640 were measured using DLS for up to 7 days at various time points (0, 1, 2, 3, and 7 day).

### Cell Culture

4T1 breast cancer cells were used for all *in vitro* experiments and were cultured in RPMI 1640 supplemented with 10% FBS and 1% P/S. Prior to the experiment, the cells were incubated at 37 °C in a 5% CO_2_ atmosphere.

### *In Vivo* Tumor Model

The 4T1 tumor models were established by a subcutaneous injection of 1 × 10^6^ cells suspended in 20 μL of PBS in the right thigh of BALB/c-nude mice. After 10 days, the tumors were confirmed to have reached a diameter of 1 cm for use in *in vivo* experiments.

### Optimization of PEG Ratio

To optimize the lipid ratio of the liposome, we compared the cellular uptake and tumor-targeting efficiency of particles formulated with different lipid ratios. Ultrasmall AuNPs loaded liposomes were synthesized using varying ratios of DSPC:DSPE-PEG5000. The amount of DSPE-PEG5000 was adjusted to five levels: absent, 0.5, 1, 2, and 4 times (0, 0.5×, 1×, 2×, 4× PEG) of the initially synthesized quantity, with 1× corresponding to 15 mol % of the total lipid. To maintain the consistent overall mole number of lipids, the amount of DSPC was adjusted correspondingly as the amount of DSPE-PEG5000 was varied.

4T1 cells were incubated with DiD-labeled Au-Lipo for 1 h, followed by washing with PBS. For nucleus staining, Hoechst 33342 was diluted in 1:2000 ratio with PBS and incubated for 5 min. Cellular uptake imaging was conducted using CLSM. The 4T1 tumor models were intravenously injected with a DiR fluorescence dye-labeled Au-Lipo and fluorescence imaging was done immediately and 24 h after injection under anesthetized with isoflurane using IVIS. Major organs and tumors were obtained at 24 h after injection for ex vivo fluorescence imaging.

### Cellular Uptake Test and *In Vitro* Cytotoxicity of Au-Lipo

Cellular uptake was observed through CLSM and cell viability was assessed using the MTT assay. For determination of cellular uptake, 4T1 cells were seeded at a density of 5 × 10^4^ cells in 35 mm confocal dishes, and DiD fluorescent dye-labeled Au-Lipo was added and incubated for 0, 2, 4, and 6 h. After PBS washing and nucleus staining with Hoechst 33342, fluorescence imaging was performed.

To evaluate the cytotoxicity of Au-Lipo, 1 × 10^4^ cells were cultured in a 96 well microplate for 24 h. Incremental concentrations (0–190 ug/mL of gold concentration) of Au-Lipo were added and incubated for 6 h. After this, MTT solution was added, and the cells were incubated for an additional 4 h. The resulting crystals were then dissolved in DMSO, and the absorbance at 540 nm was measured by using a microplate reader.

### *In Vitro* Radiosensitization Effect of Au-Lipo

To evaluate the *in vitro* radiosensitization efficiency of Au-Lipo, 4T1 cells were seeded at a density of 5 × 10^4^ cells in 35 mm culture dishes and cultured for 24 h. After 6 h of incubation with either Au-Lipo or AuroVist (190 ug/mL gold concentration), X-ray irradiation was conducted at 150 kV with different doses. Following irradiation, clonogenic assays were performed. Irradiated cells (0, 1, 2, 4, 6 Gy) were cultured for 2 weeks and then stained with crystal violet for quantification of colony formation. The number of stained colonies was quantified by using image J software. Survival curves were drawn by calculating the surviving fraction (SF) using the according to eq 2:^33^

2 $$\mathrm{SF}=\frac{N_{\mathrm{aftertreatment}}}{N_{\mathrm{cellsseeded}}\times \mathrm{PE}}$$

where *N*_aftertreatment_ is the number of colonies formed after 2 weeks in treatment group, *N*_cellsseeded_ is the initial number of cells seeded, and PE is the ratio of the number of colonies formed to the initial number of cells seeded, as derived from eq 3.

3 $$\mathrm{PE}\left(\%\right)=\frac{N_{\mathrm{beforetreatment}}}{N_{\mathrm{cellseeded}}}\times 100$$

where *N*_beforetreatment_ is the number of colonies formed after 2 weeks in the control group.

The DEF_2Gy_ values were calculated using the CFAssay package in R.

Also, X-ray-irradiated cells were stained with Annexin V-FITC and PI to perform apoptosis analysis through flow cytometry. The degree of reactive oxygen species (ROS) production and DNA double-strand breakage was evaluated by using the CellROX ROS detection reagent and γ-H2AX antibody with CLSM.

### *In Vivo* PET Imaging

^64^Cu-labeled Au-Lipo were intravenously injected into 4T1 tumor-bearing BALB/C-nude mice. Subsequently, PET was performed at various times (0, 1, 4, 24, 48 h after injection). The MIM program was used to calculate the half-life of Au-Lipo in blood circulation and its uptake in major organs (heart, liver, and spleen) and the tumor.

### *In Vivo* Radiosensitization Efficiency

To confirm the therapeutic effect of Au-Lipo in the 4T1 tumor model, Au-Lipo was intravenously injected at a dose of 300 μg of Au per mouse 10 days after the tumor model was established. Radiotherapy was then performed using 6 Gy of X-ray irradiation at 150 kV, 24 h after Au-Lipo injection. The radiosensitization efficiency was evaluated by measuring the tumor size and the weight every 2 days for 20 days following radiotherapy.

## Results and Discussion

### Characterization of Au-Lipo and Its Comparison with AuroVist

About 60 nm sized Au-Lipo was confirmed to encapsulate the ultrasmall AuNPs through TEM imaging (Figure 1A). The hydrodynamic sizes of ultrasmall AuNPs and Au-Lipo were 3.62 ± 0.74 and 65.72 ± 21.32 nm, respectively, while AuroVist exhibited a size of 1.65 ± 0.33 nm, which aligns with the specifications provided by the vendor. Bare liposomes showed a similar size to Au-Lipo (Figure 1B). The zeta potentials of AuNPs, Au-Lipo, and bare liposomes were −31.80 ± 10.50, −27.47 ± 7.26, and −19.80 ± 6.93, respectively (Figure 1C). It can be speculated that the more negative ζ-potential of Au-Lipo compared to bare liposomes is due to the incorporation of AuNPs into the liposomal membrane.^34,35^ Collectively, these results confirm the successful integration of ultrasmall AuNPs into Au-Lipo.

**Figure 1:**
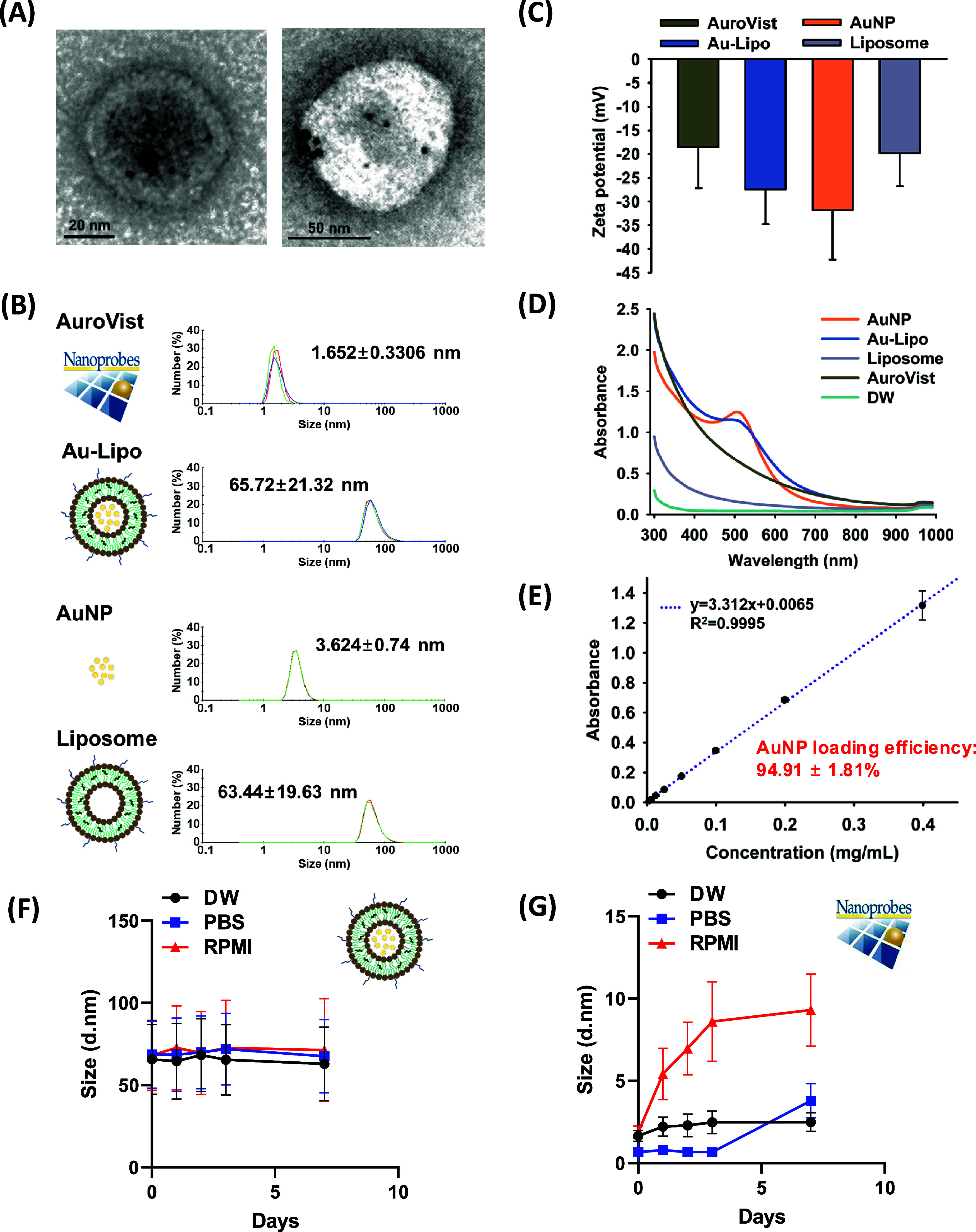
Characterization of Au-Lipo and ultrasmall AuNPs. (A) TEM image confirming the encapsulation of ultrasmall AuNPs. (B) DLS analysis showing the hydrodynamic sizes of ultrasmall AuNPs, Au-Lipo, AuroVist, and bare liposome. (C) ζ-potential measurements of AuNPs and Au-Lipo, indicating the coexistence of AuNPs and liposome based on the difference between the ζ-potential of Au-Lipo and bare liposome. (D, E) Loading efficiency of ultrasmall AuNPs in liposome, calculated using absorbance at 509 nm and a standard curve. (F, G) 7-day stability test for Au-Lipo and AuroVist in DW, PBS, or RPMI. TEM, transmission electron microscopy; AuNPs, gold nanoparticles; AuroVist, commercial AuNPs; DLS, dynamic light scattering; Au-Lipo, AuNPs encapsulated PEGylated liposome; PEG, polyethylene glycol; DW, distilled water; PBS; phosphate-buffered saline; RPMI; cell culture medium.

The loading efficiency of ultrasmall AuNPs in the liposome was determined by measuring the absorbance of AuNPs at 509 nm. The concentration of AuNPs in Au-Lipo was determined from a standard curve created by plotting the absorbance of known AuNPs concentrations, along with the initial amount of added gold. This yielded a loading efficiency of 94.91 ± 1.81% (Figure 1D,E). Accordingly, based on the measured amount of gold loaded, AuNP size, and liposome concentration measured by NTA, the average number of AuNPs encapsulated per liposome was estimated to be approximately 82. A detailed stepwise calculation is provided in Supporting Section 1. In the 7-day stability test, Au-Lipo maintained a consistent hydrodynamic size across all time points and in various solvents, indicating good colloidal stability, while AuroVist showed noticeable size variation in PBS and cell culture medium (Figure 1F,G). These findings are further supported by the DLS profiles (Figure S1) and the summarized size and PDI values (Table 1). Additionally, to assess the potential leakage of free AuNPs from Au-Lipo during storage, a control experiment was conducted by using a physical mixture of free AuNPs and empty liposomes. As expected, this mixture yielded two distinct peaks corresponding to each component (Figure S2), validating that unencapsulated AuNPs are readily detectable by DLS. Therefore, the absence of a separate AuNP peak in the Au-Lipo samples indicates that the nanoparticles remained stably encapsulated throughout the 7-day storage period.

**Table 1:**
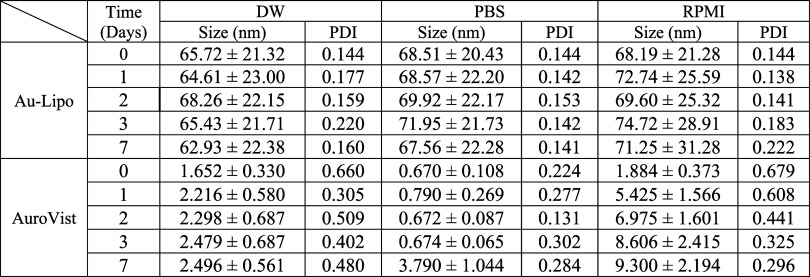
Size and Polydispersity Index (PDI) of Au-Lipo and AuroVist in DW, PBS, or RPMI^a^

a DW; distilled water, PBS; phosphate-buffered saline, RPMI; cell culture medium.

### Optimization of Au-Lipo through Adjustment of PEG Ratios and the Cytotoxicity Test

PEGylation is used as a common biocompatible and hydrophilic surface functionalization method of nanoparticles as PEG moiety expand circulation in blood by preventing eaten up from the mononuclear phagocyte system to some degree.^36–38^ However, excessive PEGylation can reduce cellular uptake by interfering with efficient interactions between nanoparticles and the cell membrane.^36,39,40^ Therefore, it is essential to find an optimal PEG concentration that strikes a balance between a prolonged circulation time and enhanced cellular uptake.

Fluorescence imaging with a confocal microscope revealed that the Au-Lipo formulated with a 1× PEG ratio demonstrated the highest cellular uptake among the concentrations of 0.5×, 1×, 2×, and 4× PEG (Figure 2A). In *in vivo* experiments, it was observed that liposomes lacking PEG components were retained by the liver immediately following intravenous injection. Besides, *in vivo* fluorescence imaging at 24 h postinjection revealed that within the PEG concentration range of 0, 0.5×, 1×, and 2×, higher PEG concentrations correlated with increased blood circulation time (Figure 2B). Major organs and tumors were harvested 24 h postinjection, and fluorescence signals were measured. The fluorescence intensity was quantified using Living Image software to calculate the tumor-to-major organ (liver, spleen, lung) uptake ratio. There was no statistically significant difference in the tumor-to-liver uptake ratio between the 1× PEG and 2× PEG formulations. Interestingly, 2× PEG showed the highest tumor-to-spleen ratio compared to the other PEG ratio. No significant difference in the tumor-to-lung ratio was observed across these concentrations (Figure 2C,D). In summary, among the varying PEG concentrations, the initially developed Au-Lipo formulation demonstrated an optimal balance of high cancer cell uptake and sufficient *in vivo* circulation.

**Figure 2:**
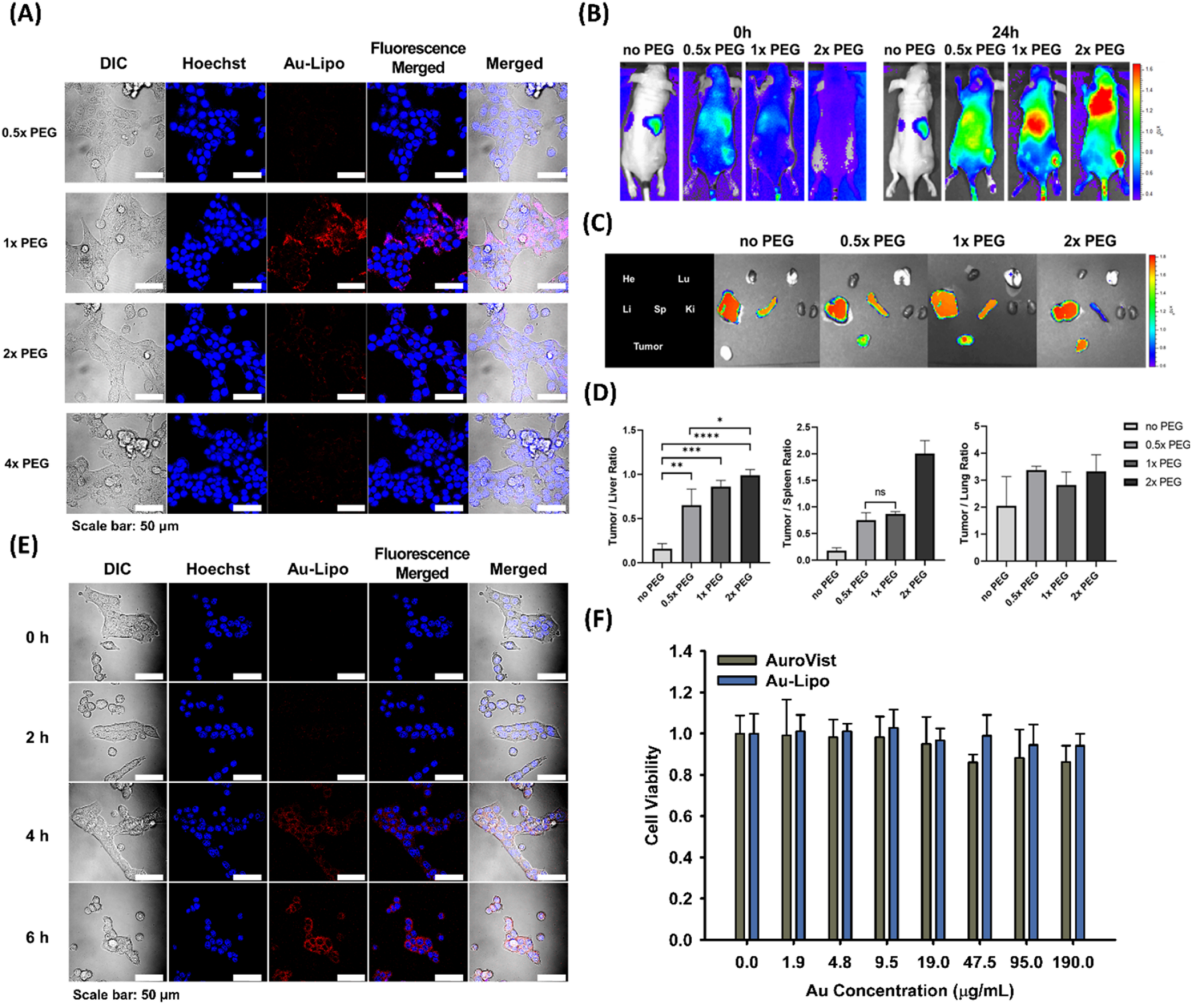
*In vitro* and *in vivo* analysis of Au-Lipo with different PEG ratios. (A) Confocal fluorescence imaging to assess cellular uptake of Au-Lipo with different PEG ratios. (B) *In vivo* fluorescence imaging at 24 h postinjection to evaluate the circulatory ability of Au-Lipo based on PEG ratio. (C) Fluorescence signal quantification from major organs (liver, spleen, lung) and tumors at 24 h postinjection to compare uptake. (D) Tumor-to-organ uptake ratio analysis based on fluorescence signal measurements (*n* = 3). (E) *In vitro* cellular uptake analysis of Au-Lipo in 4T1 cells using fluorescence imaging over a 6-h period. (F) *In vitro* cytotoxicity of Au-Lipo in 4T1 cells evaluated by MTT assay at varying concentrations of gold. * *P* < 0.05, ** *P* < 0.01, *** *P* < 0.001, **** *P* < 0.0001, AuroVist, commercial AuNPs; Au-Lipo, AuNPs encapsulated PEGylated liposome; PEG, polyethylene glycol; MTT, 3-[4,5- dimethylthiazol-2-yl]-2,5-diphenyltetrazolium bromide.

Based on the results of cellular uptake efficiency of Au-Lipo, maximum cellular uptake was observed through confocal microscopy at 6 h post-treatment. (Figure 2E). Additionally, Au-Lipo showed no significant cytotoxicity in 4T1 cells without radiation, even at a concentration of Au up to 190 μg/mL (Figure 2F). Given the results from the above experiments, subsequent *in vitro* studies were conducted with an Au concentration of 190 μg/mL and a cellular uptake time of 6 h, as this condition provided optimal efficacy with no significant cytotoxicity.

### Evaluation of the Radiosensitization Effects of Au-Lipo through *In Vitro* Tests

The radiosensitization effect of Au-Lipo can be attributed to the generation of ROS by X-ray irradiation. Gold nanoparticles have been shown to produce ROS through the interaction of high-Z gold atoms with X-rays, leading to the formation of secondary electrons that induce oxidative stress within cells.^41^ These ROS are highly cytotoxic and can cause significant DNA damage, particularly DNA double-strand breaks, which are critical for inducing cell death.^42^

The underlying mechanism supports the *in vitro* evaluation of radiosensitization efficiency of Au-Lipo under external X-ray irradiation, which was further investigated through assays measuring colony formation, apoptosis, ROS generation, and DNA double-strand breaks. The results showed that colony formation decreased progressively with increasing doses of X-ray irradiation. Furthermore, when radiation was combined with gold nanoparticle treatment, an even greater reduction in colony formation was observed, indicating the enhanced effect of the combined therapy. The DEF_2Gy_ value was calculated as 1.79 ± 0.57 for AuroVist and 2.56 ± 0.31 for Au-Lipo^43–45^ (Figure 3A–C). An apoptosis assay using flow cytometry with Annexin V-FITC and PI staining revealed that the higher mean apoptosis rate in irradiated cells treated with Au-Lipo was 11.66%, compared to a lower rate of 5.49% in cells without Au-Lipo (Figure 3D,E, Table S1). ROS production was also higher in the group treated with both Au-Lipo irradiation and X-ray irradiation than in the group treated with X-ray irradiation alone (Figure 3F). The level of DNA double-strand breaks was approximately four times higher in the group treated with both Au-Lipo and X-ray irradiation compared to X-ray irradiation alone (*P* < 0.05) (Figure 3G,H). These results demonstrate that the combination of Au-Lipo and X-ray irradiation effectively induces cancer cell death through enhanced ROS generation and DNA double-strand breaks.

**Figure 3:**
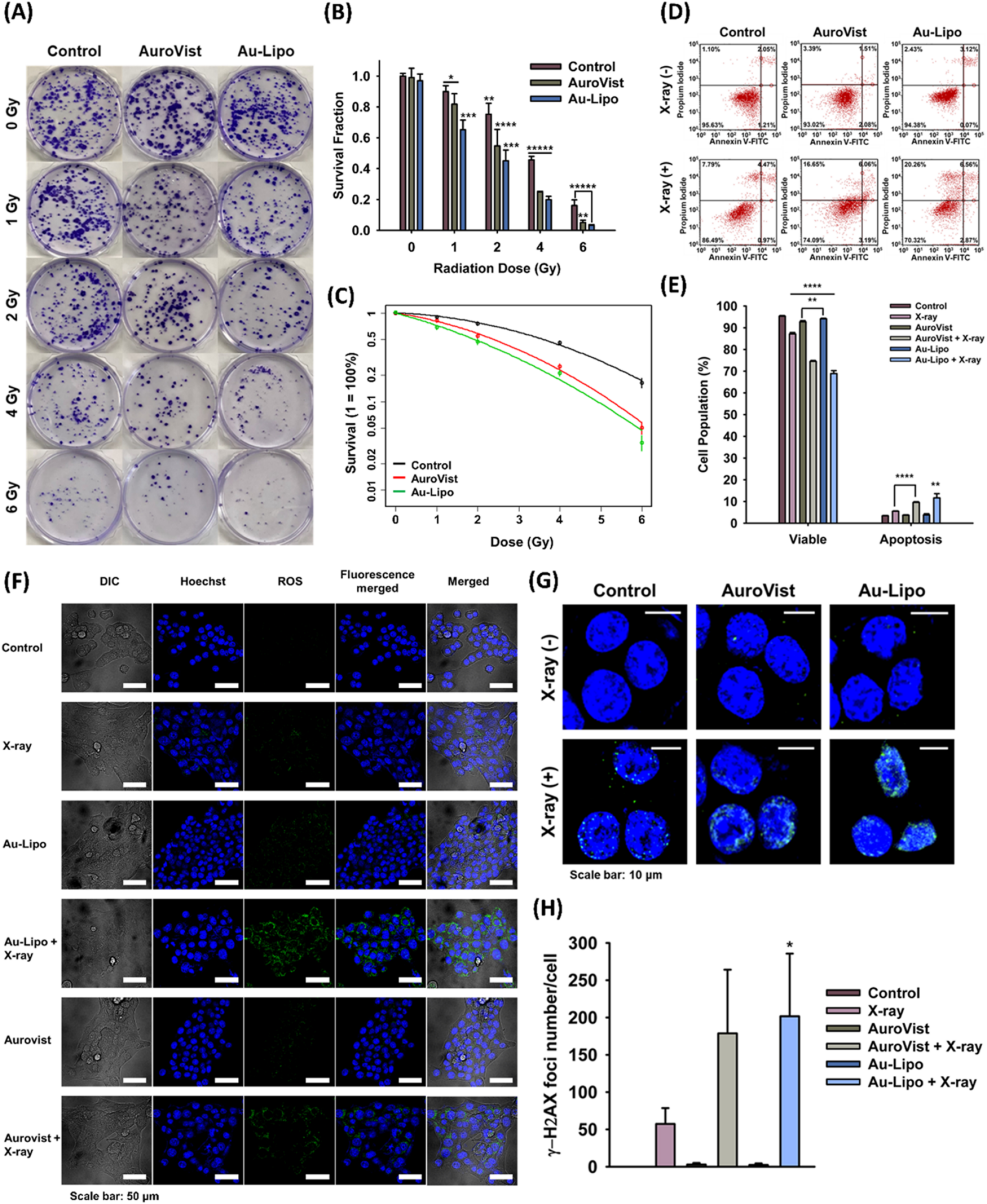
*In vitro* radiosensitization efficiency of Au-Lipo (A–C) Clonogenic assay and survival fraction of 4T1 cells to evaluate the radiosensitization effect of Au-Lipo and AuroVist, with DEF_2Gy_ (*n* = 4, 2.56 ± 0.31 for Au-Lipo, 1.79 ± 0.57 for AuroVist). (D, E) Apoptosis analysis by flow cytometry using Annexin V-FITC/PI staining to measure apoptosis rate in cells treated with Au-Lipo and X-ray irradiation (*n* = 3). (F) ROS detection assay to compare ROS detection assay with the CellROX green reagent to compare ROS generation in cells treated with X-ray alone and X-ray combined with Au-Lipo. (G, H) Immunofluorescence staining to assess DNA double-strand breaks (γ-H2AX foci) in cells after treatment with Au-Lipo and X-ray irradiation (*n* = 3). * *P* < 0.05, ** *P* < 0.01, *** *P* < 0.001, **** *P* < 0.0001, ***** *P* < 0.00001, AuroVist, commercial AuNPs; Au-Lipo, AuNPs encapsulated PEGylated liposome; PEG, polyethylene glycol; DEF_2Gy_, dose enhancement factor at 2 Gy; ROS, reactive oxygen species.

Notably, Au-Lipo demonstrated slightly higher radiosensitization efficiency than that of AuroVist. While the *in vitro* results showed only a slight advantage, the inherent properties derived from the liposome delivery platform, including prolonged circulation and remarkable tumor-targeting efficiency,^46^ suggest that *in vivo* performance of Au-Lipo could be significantly superior to that of AuroVist.

### Assessment of ^64^Cu-Labeled Au-Lipo for Biodistribution and Irradiated Au-Lipo for Therapeutic Efficacy

^64^Cu radiolabeled formulation of Au-Lipo provided insights into blood circulation and tumor-targeting efficiency, with a circulatory half-life of 11.39 h. Additionally, tumor uptake reached 20%ID/g at 24 h postintravenous injection, highlighting its suitability for enhanced tumor targeting and retention (Figure S3A–C). As shown in the results (Figure S3D), the tumor-to-major organ ratios were calculated, with particular attention to the tumor-to-liver uptake ratio, which was about 0.47 at 24 h.

Studies on the biodistribution of gold-containing nanoparticles for cancer therapy were reviewed, as summarized in Table 2. Chen et al. reported PEGylated gold nanostars (pAuNSs) with high photothermal conversion efficiency, a half-life of 2.06 h, a tumor uptake of 0.99 ± 0.38%ID/g, and a tumor-to-liver ratio of 0.073 ± 0.029.^47^ Ding et al. engineered ultrasmall, peptide-coated gold nanoparticles (Au@Tat-R-IK NPs) with a tumor uptake of 2.7%ID/g and a tumor-to-liver ratio of 0.18, enhancing radiotherapy efficacy.^48^ Wei et al. developed urchin-like gold nanoparticles (Au-Ur@DTTC) for intraoperative tumor detection, achieving a half-life of 14.22 ± 0.25 h, a tumor uptake of 4.87 ± 1.73%ID/g, and a tumor-to-liver ratio of 0.24, enhancing imaging and therapeutic precision.^49^ Yang et al. created resveratrol-coated gold nanoflowers (Au@Res NFs) for CT imaging and therapy, with a half-life of 3.01 ± 0.17 h, a tumor uptake of 6.13 ± 0.34% ID/g, and a tumor-to-liver ratio of 0.27, supporting effective tumor localization and treatment.^50^ Han et al. developed a gold nanoparticles coated with PEG-grafted polyhethylenimine with retinoic acid and small interfering RNA (Au@PP/RA/siRNA) system to enhance chemotherapy in pancreatic tumors, exhibiting a half-life of 6.21 h, a tumor uptake of 5%ID/g, and a tumor-to-liver ratio of 0.14.^51^ Sun et al. introduced chelator-free ^64^Cu-integrated gold nanomaterials (Au NMs) for PET imaging and photothermal therapy, achieving a tumor uptake of 7.6%ID/g, a tumor-to-liver ratio of 0.5, and a half-life of 17.6 h.^52^

**Table 2:** Biodistribution Properties of Various Gold-Containing Nanoparticles from Previous Studies^a^

particle name (by the authors)	half-life (hours)	tumor %ID/g (%)	tumor-to-liver ratio	refs
pAuNSs	2.06	0.99	0.073	^47^
Au@Tat-I-EK	not specified	2.7	0.18^b^	^48^
Au-Ur@DTTC	14.22	4.87	0.24^b^	^49^
Au@Res NFs	3.01	6.13	0.27^b^	^50^
Au@PP/RA/siRNA	6.21	5^b^	0.14^b^	^51^
Au NMs	17.6	7.6	0.35^b^	^52^
Au-Lipo	11.39	20	0.47	this study

a pAuNSs, PEGylated gold nanostars; Au@Tat-I-EK, peptide-coated ultrasmall gold nanoparticles; Au-Ur@DTTC, urchin-like gold nanoparticles; Au@Res NFs, resveratrol-coated gold nanoflowers; Au@PP/RA/siRNA, gold nanoparticles coated with PEG-grafted polyhethylenimine with retinoic acid and small interfering RNA; Au NMs, chelator-free ^64^Cu-integrated gold nanomaterials; Au-Lipo, ultrasmall AuNPs loaded PEGylated liposome; PEG, polyethylene glycol.

b Value directly calculated from the graph in the ref.

In this study, Au-Lipo demonstrated a tumor uptake of 20%ID/g, a tumor-to-liver ratio of 0.47, and a half-life of 11.39 h, suggesting favorable biodistribution, *in vivo* stability, and circulation time compared to the findings reported in prior studies on gold-containing nanoparticles. The remarkable tumor accumulation of AuNPs observed in this study may be attributed to optimization of the PEG ratio applied in the PEGylation of liposomes. Increasing the PEG surface density on nanoparticles is known to prolong circulation time in blood; however, excessive amounts of PEG used in nanoparticle formulation do not necessarily guarantee greater surface coverage.^37^ Thus, optimizing the PEG-lipid conjugate ratio is crucial to achieving a balance between cost-effectiveness and a sufficient *in vivo* circulation capability. Additionally, cellular uptake results (Figure 2A) indicate that overly high PEG ratios significantly reduce cellular uptake. This finding aligns with the study by Hak et al., which demonstrated an inverse relationship between PEG ratio and cellular uptake at higher PEG levels.^53^ Based on the combined *in vivo* and *in vitro* results, the optimized PEG ratio in this study likely plays a critical role in achieving the identified significant tumor accumulation.

This favorable outcome is further underscored when considered alongside previously studied ultrasmall gold nanoparticles such as AuroVist which exhibit rapid renal clearance and limited tumor accumulation. These characteristics result from their ultrasmall hydrodynamic sizes, which are suitable for renal imaging or rapid clearance but less appropriate for tumor-targeted radiosensitization that requires prolonged circulation and EPR-based accumulation. For instance, AuroVist demonstrates tumor accumulation of 4.9 ± 0.6% ID/g at 5 min postinjection, with a tumor half-life of approximately 40 min.^25^ Despite AuroVist and Au-Lipo showed comparable radiosensitizing effects *in vitro*, Au-Lipo achieved significantly improved tumor retention *in vivo*, attributed to its PEGylated liposomal encapsulation. Given the well-established pharmacological behavior of these ultrasmall gold nanoparticles, our *in vivo* therapeutic investigation focused on Au-Lipo, which presented a more favorable biodistribution profile and higher translational relevance for radiotherapy applications.

To further assess this system, we evaluated the *in vivo* radiosensitization efficiency of Au-Lipo by treating 4T1 tumor-bearing mice with X-ray irradiation with or without Au-Lipo. Results demonstrated that the combination of Au-Lipo and X-ray provided the most effective treatment outcome, showing enhanced efficacy as a radiation sensitizer compared to radiotherapy alone, with no significant difference in mouse body weight (Figure 4A–C).

**Figure 4:**
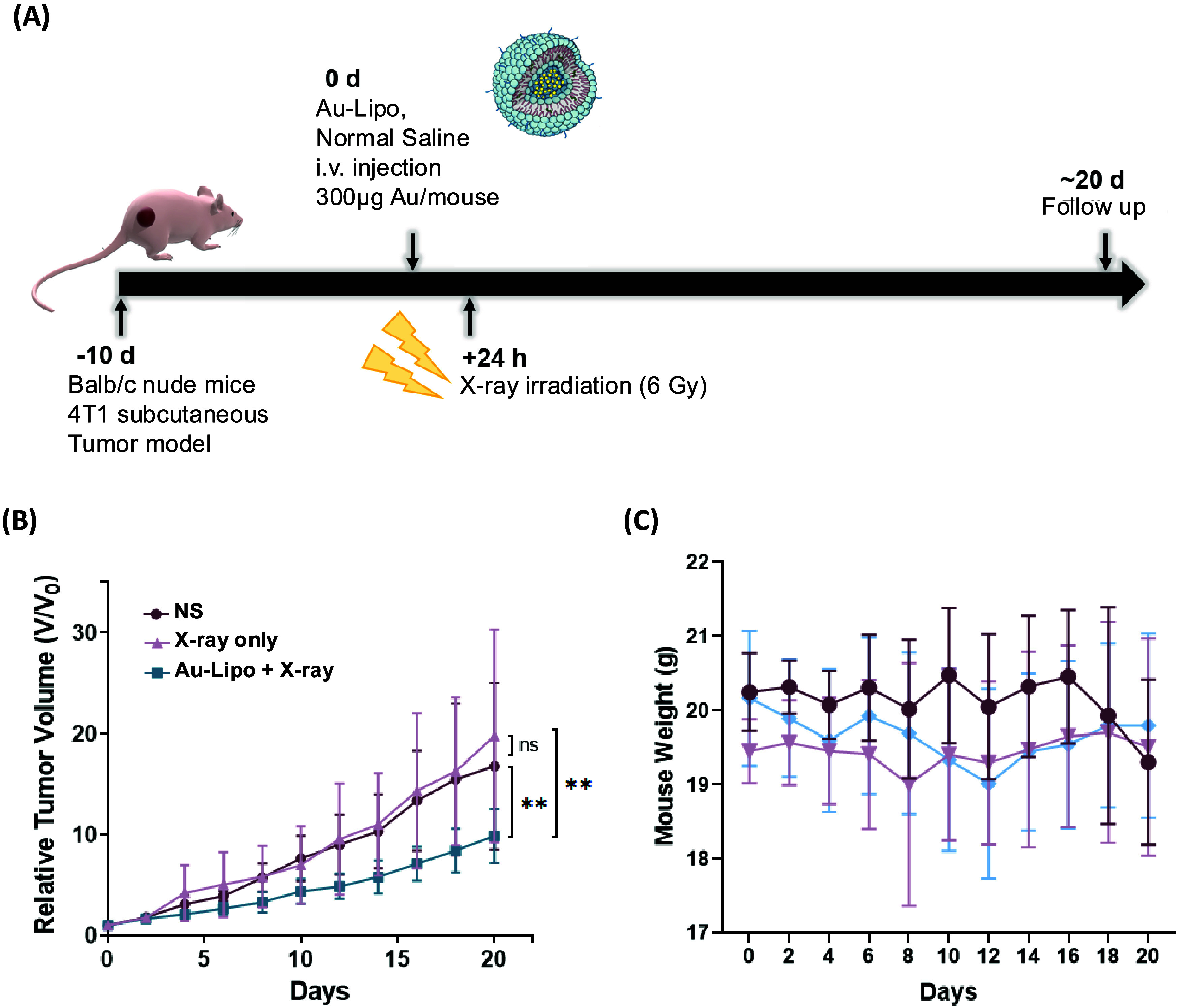
Radiosensitization efficiency of Au-Lipo. (A) Schematic illustration of radiotherapy plan utilizing Au-Lipo and X-ray irradiation. (B, C) *In vivo* radiosensitization efficiency of Au-Lipo in a 4T1 mouse tumor model, treated with or without X-ray irradiation, showing enhanced therapeutic efficacy of Au-Lipo combined with radiotherapy without affecting mouse body weight (*n* = 5). ** *P* < 0.01, Aurovist, commercial AuNPs; Au-Lipo, AuNPs encapsulated PEGylated liposome; PEG, polyethylene glycol.

Notably, X-ray irradiation alone at 6 Gy did not induce significant tumor suppression compared with the untreated control group. This may be partially attributed to the intrinsic radioresistance of the 4T1 tumor model^54,55^ and the limited biological impact of a single-dose irradiation protocol. Several studies have reported improved therapeutic outcomes when fractionated regimens were used, as repeated radiation exposure can lead to cumulative DNA damage and sustained activation of antitumor immune responses.^56,57^ Consistent with our results, other studies have also reported insignificant tumor inhibition compared to control groups following radiotherapy alone,^58,59^ indicating that such outcomes can vary depending on the tumor model and experimental conditions. These findings underscore the necessity of combination approaches for enhancing the therapeutic efficacy.

## Conclusions

In this study, we successfully synthesized and optimized Au-Lipo, a PEGylated liposome loaded with ultrasmall AuNPs, which exhibited excellent stability, biocompatibility, and tumoral retention by optimizing the PEGylated lipid ratio and also demonstrated high radiosensitization efficacy. *In vitro* experiments confirmed the ability of Au-Lipo to generate ROS under X-ray irradiation, leading to significant cancer cell killing. *In vivo* evaluations further demonstrated that Au-Lipo possesses extended blood circulation and superior tumor accumulation, which collectively contribute to its pronounced therapeutic efficacy. Notably, Au-Lipo achieved these outcomes without noticeable adverse effects, highlighting its potential as a highly biocompatible and tumor-targeting radiosensitizer for advanced cancer therapy.

## Supplementary Material



## Notes

Detailed estimation of the number of AuNPs per liposome based on characterization data; stability analysis of Au-Lipo and AuroVist; DLS profile of free AuNPs mixed with empty liposomes; flow cytometry-based apoptosis analysis; and biodistribution of ^64^Cu-labeled Au-Lipo with tumor-to-organ ratios and circulation kinetics (PDF)

## Abbreviations

Au: gold
AuroVist: commercial AuNPs
AuNPs: gold nanoparticles
PEG: polyethylene glycol
Au-Lipo: ultrasmall AuNPs loaded PEGylated liposome
DEF_2Gy_: dose enhancement effect factor at 2Gy
HAuCl_4_: gold­(III) chloride trihydrate
NaOH: sodium hydroxide
THPC: tetrakis hydroxymethyl phosphonium chloride
DiD: 1, 1′-dioctadecyl-3, 3, 3′, 3′-Tetramethylindodicarbocyanine
DiR: 1, 1′-dioctadecyl-3, 3, 3′, 3′-Tetramethylindotricarbocyanine iodide
MTT: 3-[4,5- dimethylthiazol-2-yl]-2,5-diphenyltetrazolium bromide reagent
DSPC: 1,2-distearoyl-*sn*-glycero-3-phosphocholine
DSPE-PEG2000-amine: 1,2-distearoyl-*sn*-glycero-3-phosphoethanolamine-N-[amino­(polyethylene glycol)-2000]
DSPE-PEG5000: 1,2-distearoyl-*sn*-glycero-3-phosphoethanolamine-N-[methoxy­(polyethylene glycol)-5000]
p-SCN-Bn-NOTA: 2-(p-Isothiocyanatobenzyl)-1,4,7-triazacyclononane-N,N′,N,″-triacetic acid trihydrochloride
FBS: fetal bovine serum
P/S: penicillin-streptomycin
PBS: phosphate-buffered saline
DW: distilled water
DMSO: dimethyl sulfoxide
TEM: transmission electron microscope
DLS: dynamic light scattering
NTA: nanoparticle tracking analyzer
CLSM: confocal laser scanning microscope
PET: positron emission tomography
ROS: reactive oxygen species
s.d.: standard deviation
P: probability value
pAuNSs: PEGylated gold nanostars
Au@Tat-R-IK NPs: peptide-coated gold nanoparticles
Au-Ur@DTTC: urchin-like gold nanoparticles
Au@Res NFs: resveratrol-coated gold nanoflowers
Au@PP/RA/siRNA: gold nanoparticles coated with PEG-grafted polyhethylenimine with retinoic acid and small interfering RNA
Au NMs: chelator-free ^64^Cu-integrated gold nanomaterials
