# Supplementary material for: Theranostic Gold Nanoparticles Encapsulated in a PEGylated Liposome as an Effective Radiosensitizer for Cancer Radiation Therapy
Source: ACS Appl Bio Mater. 2025 Aug 26;8(9):7877–88. doi: 10.1021/acsabm.5c00908 (PMC12442100; doi:10.1021/acsabm.5c00908)
Supplement: Supplementary file 1 [file mt5c00908_si_001.pdf]

Supporting Information

Theranostic Gold Nanoparticles Encapsulated in PEGylated Liposome as Effective Radiosensitizer for Cancer Radiation Therapy

Jinyeong Choi[1,5], Gaeun Kim[1,5], Beomjin Park[2,5], Jiwoo Park[2], Shengjun Li[2], Wooseung Lee[1], Miyeon Jeon[1], Chiwoo Oh[1], Sangmin Lee[1], Sung-Joon Ye[1], Hyung-Jun Im[1,2,3,4*]*

*Correspondence: Hyung-Jun Im, iiihjjj@snu.ac.kr; Sung-Joon Ye, sye@snu.ac.kr

1. Department of Applied Bioengineering, Graduate School of Convergence Science and Technology, Seoul National University, Seoul, 08826, Republic of Korea

2. Department of Molecular Medicine and Biopharmaceutical Sciences, Graduate School of Convergence Science and Technology, Seoul National University, Seoul, 08826, Republic of Korea

3. Cancer Research Institute, Seoul National University, 03080 Seoul, Republic of Korea

4. Research Institute for Convergence Science, Seoul National University, Seoul, 08826, Republic of Korea

5. These authors contributed equally to this work.

Section 1. Estimation of the Number of AuNPs per Liposome

To estimate the number of AuNPs encapsulated within each liposome (Au-Lipo), we performed a stepwise calculation based on the synthesis input, particle characterization, and nanoparticle tracking analysis (NTA) data, as described below.

1) Estimation of Total AuNPs Loaded into Liposomes

① Amount of gold input

A total of 140 µL of 1% $HAuCl_4$ solution was used for AuNP synthesis, corresponding to approximately 0.812 mg of elemental gold.

② Gold loading efficiency

The gold loading efficiency was experimentally determined to be 94.91%, based on UV–vis absorbance at 509 nm (Fig. 1D–E). Thus, the total amount of gold successfully encapsulated into liposomes was calculated as:

$$0.812\ \mathrm{mg} \times 0.9491 = 0.7707\mathrm{mg} \tag{S1}$$

③ Mass of a single AuNP

Assuming spherical AuNPs with an average diameter of 3.624 nm, the volume of one particle was calculated as:

$$V = \frac{4}{3}\pi(1.812)^3 \approx 24.96\mathrm{nm}^3 \tag{S2}$$

Using the density of gold ($19.3\ \mathrm{g/cm^3} = 19.3\times10^{-21}\ \mathrm{g/nm^3}$) the mass of a single AuNP was:

$$24.96\mathrm{nm}^3 \times 19.3 \times 10^{-21}\mathrm{g/nm}^3 = 4.82 \times 10^{-19}\mathrm{g} \tag{S3}$$

④ Total number of AuNPs

Dividing the total mass of gold by the mass of one AuNP yields:

$$\frac{7.707 \times 10^{-4}\text{g}}{4.82 \times 10^{-19}\text{g}} \approx 1.599 \times 10^{15} \text{ particles} \quad \text{(S4)}$$

2) Quantification of Liposomes

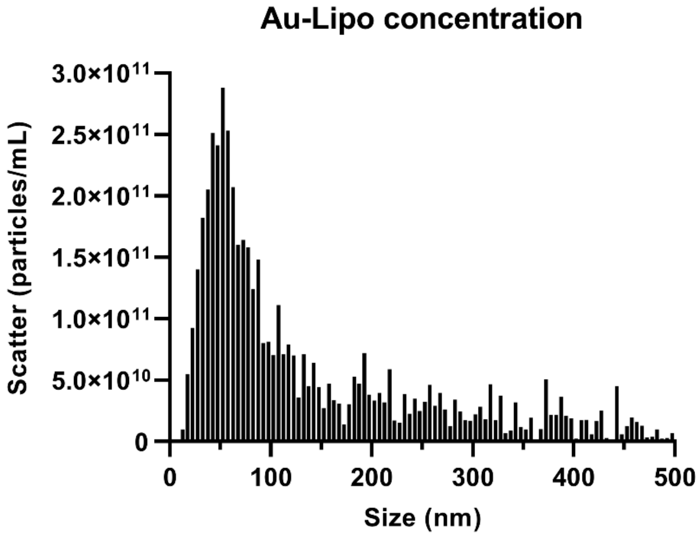

	Most frequent particle size [nm]	Concentration (particles/mL)
Au-Lipo 1	52.5	5.53×10^{12}
Au-Lipo 2	67.5	5.51×10^{12}
Au-Lipo 3	65.5	5.58×10^{12}

① Final sample volume and liposome concentration
After PD-10 purification, the final volume of Au-Lipo dispersion was 3.5 mL.

According to NTA measurements, the concentration of Au-Lipo was:

$$5.54 \times 10^{12} \text{ particles}/mL \quad \text{(S5)}$$

Therefore, the total number of liposomes was:

$$5.54 \times 10^{12} \times 3.5 = 1.939 \times 10^{13} \text{ particles} \quad \text{(S6)}$$

3) Average Number of AuNPs per Liposome

① Encapsulation ratio
The average number of AuNPs encapsulated per liposome was obtained by:

$$\frac{1.599 \times 10^{15}}{1.939 \times 10^{13}} \times 10^{13} \approx 82.5 \text{ AuNPs/Liposome} \qquad \text{(S7)}$$

This result appears to align well with the theoretical loading capacity of a liposome around 65.72 nm in diameter, supporting the feasibility and internal consistency of the loading strategy used in this study.

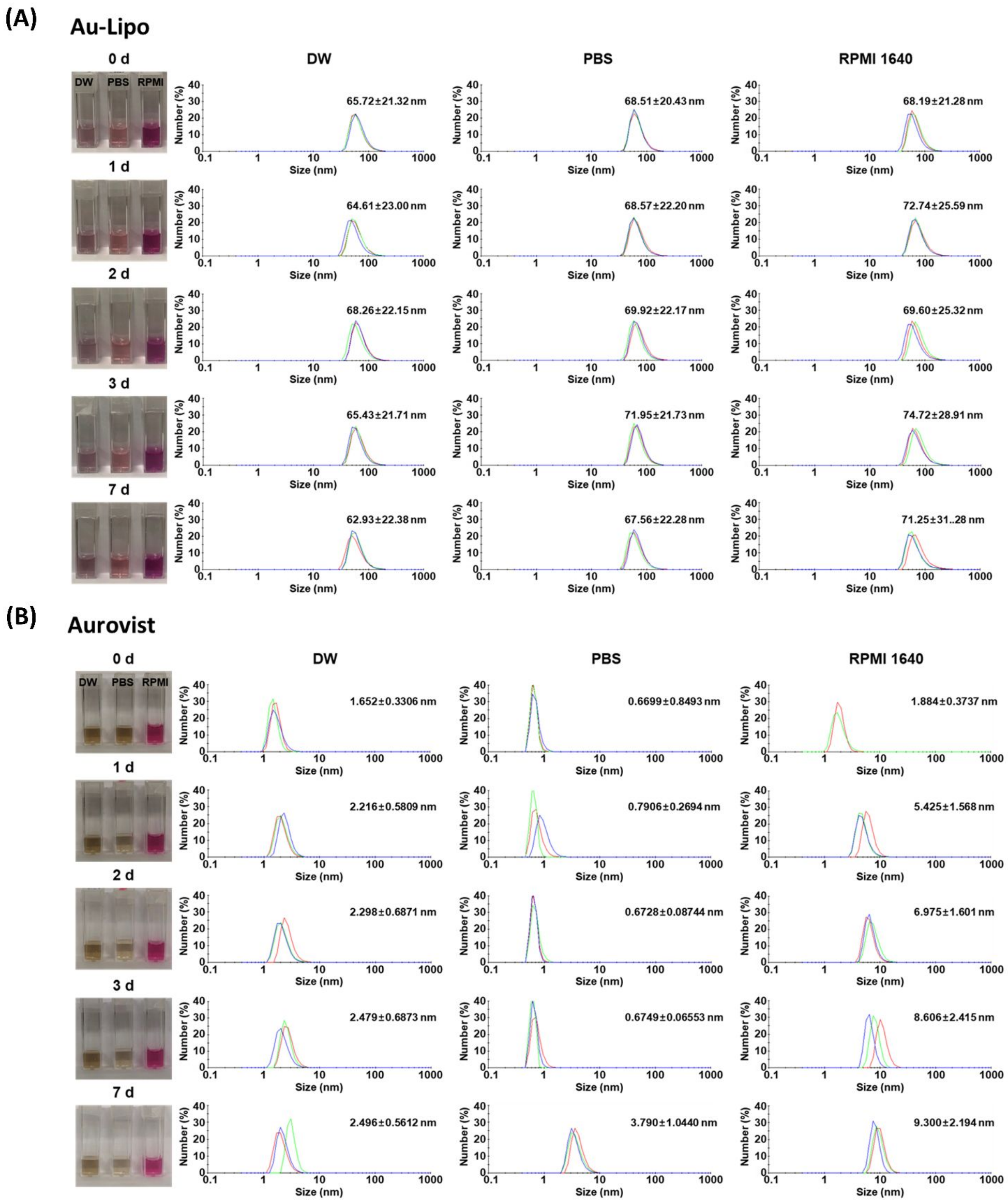

Figure S1. Stability analysis of Au-Lipo and AuroVist

(A) DLS analysis of Au-Lipo showing consistent hydrodynamic size over 7 days in different solvents. (B) Hydrodynamic size changes of AuroVist over 7 days. DLS, Dynamic light scattering;

AuroVist, commercial AuNPs; AuNP, Gold nanoparticle; Au-Lipo, AuNPs encapsulated PEGylated liposome; PEG, Polyethylene glycol.

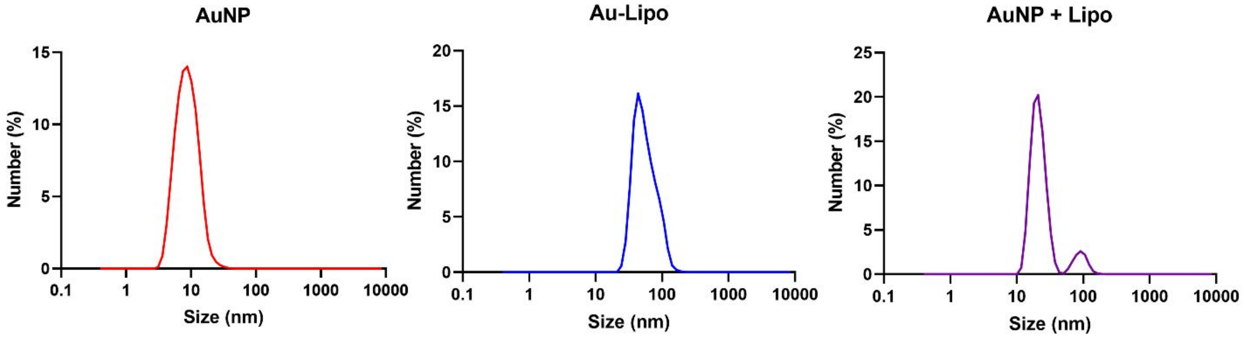

Figure S2. DLS profile of a control sample: free AuNPs mixed with empty liposomes

DLS analysis of a control mixture of free AuNPs and empty liposomes, showing two distinct peaks corresponding to unencapsulated AuNPs and liposomes, respectively.

	Control	X-ray	AuroVist	AuroVist + X-ray	Au-Lipo	Au-Lipo + X-ray
**Viable (%)**	95.31 ± 0.30	87.17 ± 0.61	92.75 ± 0.52	74.52 ± 0.42	94.13 ± 0.22	68.91 ± 1.33
**Apoptosis (%)**	3.42 ± 0.14	5.49 ± 0.18	3.71 ± 0.16	9.57 ± 0.30	3.80 ± 0.53	11.66 ± 1.95

Table S1. Apoptosis analysis by flow cytometry using Annexin V-FITC/PI staining to measure apoptosis rate in cells treated with Au-Lipo and X-ray irradiation (n = 3).

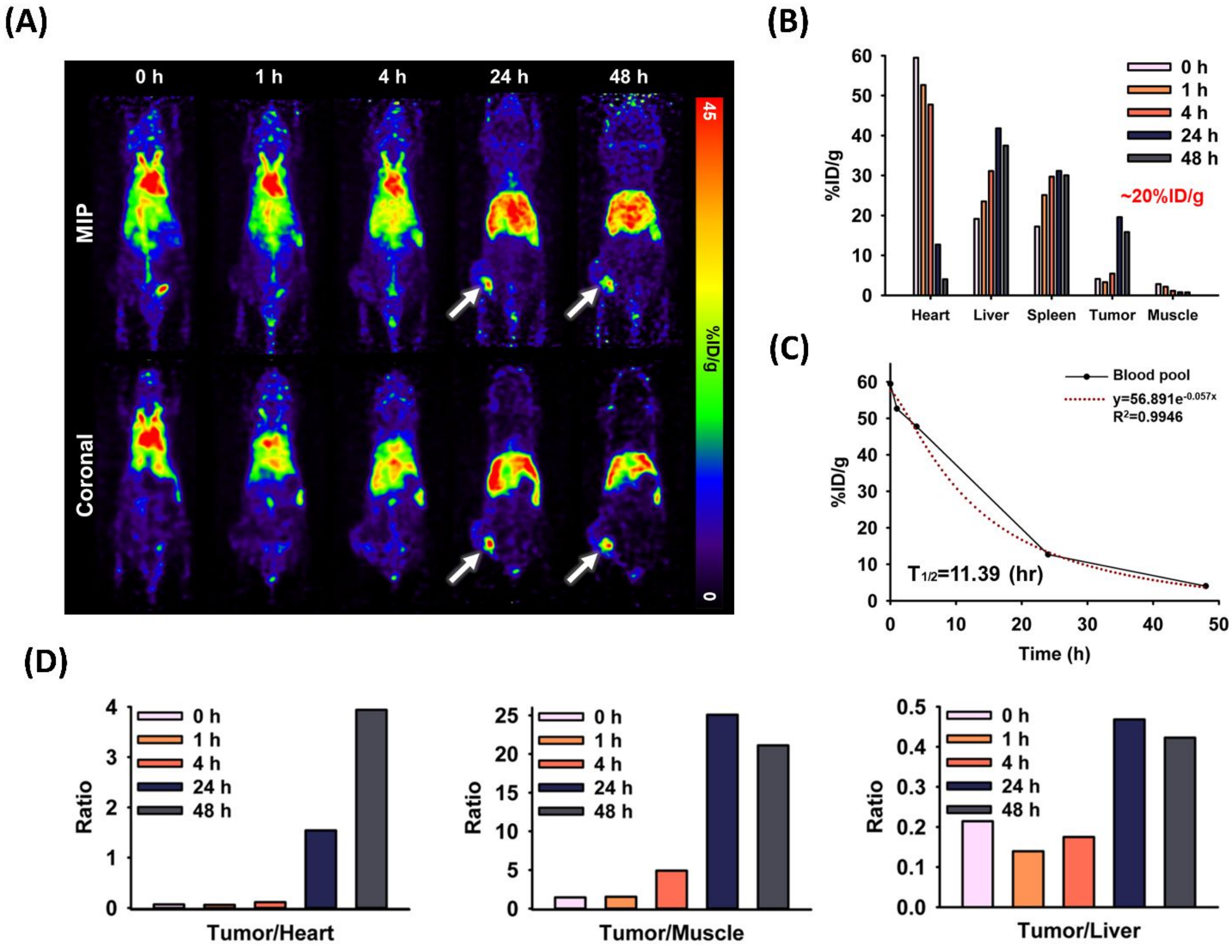

Figure S3. (A–C) Imaging and biodistribution analysis of ^{64}Cu-labeled Au-Lipo, showing blood circulation and tumor targeting efficiency, with a calculated circulatory half-life of 11.39 hours and tumor uptake of 20 %ID/g at 24 hours post-injection. (D) Tumor-to-major organ ratio at 24 hours post-injection, with a tumor-to-liver uptake ratio (n = 1). Au-Lipo, AuNP encapsulated PEGylated liposome; AuNP, Gold nanoparticle; PEG, Polyethylene glycol.
